# Alkaline Phosphatase and Cumulative Vascular Access Burden in Maintenance Hemodialysis: An Exploratory Clinical Study

**DOI:** 10.3390/jcm15218472

**Published:** 2026-10-30

**Authors:** Daniela Marinescu, Laurențiu Augustus Barbu, Tiberiu Stefăniță Țenea Cojan, Daniela-Teodora Maria, Sorin-Ioan Zaharie, Răzvan Alexandru Marinescu, Valeriu Șurlin, Daniel-Cosmin Caragea, Ana-Maria Ciurea, Romeo Popa

**Affiliations:** 1 Department of Surgery, Emergency County Hospital, University of Medicine and Pharmacy of Craiova, 2 Petru Rares Street, 200349 Craiova, Romania; dtmarinescu@yahoo.com (D.M.); vsurlin@gmail.com (V.Ș.); 2 Department of Surgery, Railway Clinical Hospital Craiova, University of Medicine and Pharmacy of Craiova, 2 Petru Rares Street, 200349 Craiova, Romania; tiberiu.tenea@umfcv.ro; 3 Department of Nephrology, Emergency County Hospital, University of Medicine and Pharmacy of Craiova, 2 Petru Rares Street, 200349 Craiova, Romania; daniela.maria@umfcv.ro (D.-T.M.); szaharie3@yahoo.com (S.-I.Z.); daniel.caragea@umfcv.ro (D.-C.C.); romeo.popa@umfcv.ro (R.P.); 4 Department of Plastic Surgery, University of Medicine and Pharmacy of Craiova, 200349 Craiova, Romania; 5 Academy of Romanian Scientists, 3 Ilfov Street, 050044 Bucharest, Romania; 6 Research Center for Minimally Invasive Surgery, University of Medicine and Pharmacy of Craiova, 2 Petru Rares Street, 200349 Craiova, Romania; 7 Department of Oncology, Emergency County Hospital, University of Medicine and Pharmacy of Craiova, 2 Petru Rares Street, 200349 Craiova, Romania; amciurea14@gmail.com

**Keywords:** alkaline phosphatase, hemodialysis, vascular access, arteriovenous fistula, CKD–MBD, vascular calcification, parathyroid hormone

## Abstract

**Background**: Vascular access dysfunction remains a major challenge in maintenance hemodialysis (HD). Although chronic kidney disease–mineral and bone disorder may contribute to vascular pathology, the relationship between mineral metabolism markers and cumulative vascular access deterioration remains poorly defined. **Methods**: This retrospective single-center study included 74 patients receiving maintenance HD. Cumulative vascular access burden was defined by reinterventions, definitive access losses, and additional vascular accesses. Associations with parathyroid hormone (PTH), calcium, phosphorus, calcium–phosphorus product, and alkaline phosphatase (ALP) were evaluated using group comparisons, correlation analysis, and logistic regression. **Results**: Patients with cumulative vascular access burden had higher ALP levels than those without burden (92.0 vs. 78.0 U/L, *p* = 0.010), whereas PTH, calcium, phosphorus, and calcium–phosphorus product did not differ significantly. ALP correlated with cumulative burden (Spearman’s ρ = 0.303, *p* = 0.009), and this association remained statistically significant after adjustment for age, BMI, PTH, and phosphorus (OR per 10 U/L increase = 1.226, 95% CI 1.059–1.418; *p* = 0.006). **Conclusions**: Higher ALP levels were associated with greater cumulative historical vascular access burden after adjustment for selected clinical and biochemical variables. These exploratory findings do not establish the prognostic value of ALP for future vascular access outcomes and require prospective validation.

## 1. Introduction

Maintaining functional vascular access is essential for patients undergoing long-term hemodialysis (HD). Arteriovenous fistulas (AVFs) are generally considered the preferred option because of their favorable long-term outcomes. However, vascular access complications remain a persistent challenge in clinical practice, frequently requiring additional procedures to manage thrombosis, restore patency, or replace failed accesses [1,2]. The consequences of vascular access dysfunction are not limited to a single complication. Over time, some patients undergo multiple interventions, experience permanent access loss, and require the creation of new vascular accesses. Together, these events contribute to a growing clinical burden throughout the course of dialysis treatment [3,4]. Considering the entire history of access-related events, rather than evaluating each complication separately, may offer a more comprehensive understanding of vascular access deterioration.

Several interconnected mechanisms contribute to vascular access dysfunction, including neointimal hyperplasia, endothelial injury, inflammatory responses, thrombosis, and alterations in vascular remodeling. In patients with advanced chronic kidney disease, these changes develop in the context of broader metabolic disturbances that may further compromise vascular health. Among these, chronic kidney disease–mineral and bone disorder (CKD–MBD) is particularly relevant because abnormalities in mineral homeostasis and bone metabolism are associated with vascular calcification and cardiovascular complications [5]. Rather than representing a passive accumulation of calcium, vascular calcification involves active biological changes, including the acquisition of bone-like characteristics by vascular smooth muscle cells. This process may help explain how CKD–MBD contributes to structural changes in blood vessels and potentially affects the long-term function of hemodialysis vascular access [5,6,7].

Although parathyroid hormone (PTH) remains a central parameter in the evaluation of chronic kidney disease–mineral and bone disorder (CKD–MBD), its circulating levels do not necessarily capture the full spectrum of changes in bone turnover or their potential vascular implications. Alkaline phosphatase (ALP), particularly the bone-specific isoenzyme, offers additional insight into bone metabolic activity and mineralization processes, with potentially less biological variability than PTH [8,9,10]. Beyond its role as a biochemical marker, ALP may participate in vascular calcification by degrading inorganic pyrophosphate, a physiological inhibitor of mineral deposition [10]. Clinical observations in hemodialysis populations have also identified associations between ALP-related bone turnover abnormalities and vascular calcification, further supporting the potential relevance of this pathway [8,11].

The clinical relevance of alkaline phosphatase (ALP) extends beyond its involvement in mineral metabolism. Previous studies have reported associations between elevated ALP concentrations and adverse outcomes, including hospitalization, cardiovascular complications, infections, and mortality, among patients with chronic kidney disease (CKD), particularly those receiving maintenance hemodialysis (HD) [10,12,13]. Moreover, ALP and parathyroid hormone (PTH) may capture different aspects of bone and vascular pathology, potentially providing complementary information beyond conventional mineral metabolism parameters [8]. Nevertheless, although ALP has been extensively studied in relation to vascular calcification and survival, considerably less is known about its association with the accumulated burden of vascular access-related events over the course of hemodialysis treatment [14].

Previous research has highlighted the relationship between alkaline phosphatase (ALP), altered mineral metabolism, and vascular calcification in patients with chronic kidney disease [8]. More recently, Si et al. identified an association between elevated preoperative ALP concentrations and failure of arteriovenous fistula maturation [15]. However, the relationship between ALP and the overall history of vascular access-related events remains less well understood, particularly when repeated procedures, permanent access losses, and the need for additional accesses are considered together. Against this background, we conducted an exploratory study to examine whether serum ALP levels were associated with cumulative vascular access burden in patients undergoing maintenance hemodialysis. We also assessed conventional markers of mineral metabolism, including PTH, calcium, phosphorus, and the calcium–phosphorus product, to provide a broader biochemical context for the observed associations.

## 2. Materials and Methods

### 2.1. Study Design and Setting

This single-center retrospective observational study included adult patients receiving maintenance hemodialysis at the Department of Nephrology, Emergency County Clinical Hospital Craiova, Romania, between 1 January 2021 and 31 December 2025. The study evaluated the relationship between routinely measured markers of mineral metabolism and cumulative vascular access burden, with particular emphasis on parathyroid hormone (PTH) and alkaline phosphatase (ALP).

Clinical, laboratory, dialysis-related, and vascular access data were retrospectively extracted from electronic and paper medical records using a predefined data collection template. During the study period, a total of 83 patients receiving maintenance hemodialysis were assessed for eligibility. Of these, 9 patients (10.8%) were excluded because of incomplete medical records that prevented reliable assessment of the variables required for the study. Consequently, 74 patients (89.2%) were included in the final analysis.

### 2.2. Study Population and Eligibility Criteria

Adult patients aged ≥18 years receiving maintenance hemodialysis during the study period were eligible for inclusion. Patients were included if essential clinical and laboratory data were available and their vascular access history could be reliably reconstructed from the available medical records. A sufficiently documented vascular access history was defined as the availability of information allowing reliable determination of the number of previous vascular access-related reinterventions, definitive access losses, and additional vascular accesses created beyond the initial access. Patients were excluded when incomplete medical documentation prevented reliable assessment of the cumulative vascular access burden or when essential clinical or laboratory variables required for the primary analyses were unavailable. The unit of analysis was the individual patient.

### 2.3. Data Collection and Study Variables

Demographic and clinical variables included age, sex, body mass index (BMI), underlying cause of chronic kidney disease, diabetes mellitus, duration of hemodialysis, dialysis adequacy expressed as Kt/V, vascular access type, access location, and access vintage.

Vascular access history included documented stenosis, thrombosis, aneurysm, access infection, the number of access-related reinterventions, definitive access losses, and the sequence of vascular access creation. Vascular access type was categorized as arteriovenous fistula (AVF), arteriovenous graft (AVG), or central venous catheter (CVC), as applicable.

Laboratory variables included PTH, serum calcium, phosphorus, total ALP, C-reactive protein (CRP), and hemoglobin. The calcium–phosphorus product (Ca × P) was calculated by multiplying serum calcium by serum phosphorus and was expressed as mg^2^/dL^2^.

### 2.4. Definition of Cumulative Vascular Access Burden

The primary outcome was cumulative vascular access burden, an exploratory patient-level measure developed to quantify the accumulated frequency of documented vascular access-related events. Three components were considered in constructing this indicator: the total number of vascular access-related reinterventions (R), the number of definitive vascular access losses (L), and the number of additional vascular accesses created beyond the initial access (A). The cumulative burden score was calculated as follows:

***Cumulative vascular access burden* = *R* + *L* + *A***

Each documented event contributed one point to the cumulative score, with equal numerical weights assigned to all three components. This unweighted approach was adopted to ensure methodological simplicity, transparency, and reproducibility, while avoiding the introduction of arbitrary severity-based weights in the absence of an established and validated weighting framework. Although reinterventions, definitive access losses, and additional vascular access creation differ in their clinical consequences, prognostic implications, and healthcare resource utilization, the indicator was designed to quantify the accumulated frequency of these events rather than their relative clinical severity. Therefore, equal numerical weighting should not be interpreted as indicating equivalent clinical importance among the individual components.

Repeated vascular access-related procedures were counted individually when they represented distinct documented reinterventions. Each definitive access loss and each additional vascular access contributed one point to the cumulative burden score. When definitive access loss was followed by the creation of a new vascular access, two points were assigned, one for each component. Underlying complications, such as thrombosis, stenosis, aneurysm, or infection, were not independently added to the score. Thus, overlapping clinical complications did not generate additional points unless they resulted in separately recorded qualifying events.

For comparative and logistic regression analyses, cumulative vascular access burden was dichotomized into the absence (score = 0) or presence (score ≥ 1) of documented events. The cumulative score was also retained as a count variable for correlation analyses. Accordingly, the proposed indicator was considered an exploratory measure of cumulative vascular access-related events rather than a validated clinical severity score.

### 2.5. Laboratory Assessment

Laboratory parameters were obtained from routine clinical monitoring records of patients receiving maintenance hemodialysis. For the present analysis, a single measurement per patient was used for each laboratory parameter, including PTH, calcium, phosphorus, total ALP, C-reactive protein (CRP), and hemoglobin. These measurements were not averaged over a predefined period, and serial biochemical measurements were not analyzed. Clinical and dialysis-related characteristics were retrospectively extracted from medical records, whereas cumulative vascular access burden was reconstructed from the documented history of vascular access-related events.

Serum PTH was determined by electrochemiluminescence immunoassay (ECLIA) using a Cobas e601 analyzer (Roche Diagnostics GmbH, Mannheim, Germany). Total serum ALP activity was measured using an automated enzymatic colorimetric method on a Cobas clinical chemistry platform (Roche Diagnostics GmbH, Mannheim, Germany). The assay measured total ALP activity rather than the bone-specific isoenzyme. Bone-specific ALP measurements and hepatobiliary biochemical parameters were not systematically available in the retrospective dataset. Consequently, the relative contributions of bone-derived and hepatic ALP could not be determined, and hepatic contributions could not be reliably excluded. Therefore, total ALP was evaluated as a routinely available biochemical parameter rather than a specific marker of bone turnover or vascular calcification. Serum calcium, phosphorus, and CRP were determined using routine automated biochemical methods, while hemoglobin was measured using an automated hematology analyzer according to institutional laboratory protocols.

The laboratory measurements represented a single biochemical assessment for each patient, whereas cumulative vascular access burden reflected the documented history of access-related events. Because laboratory measurements were not necessarily obtained before the individual vascular access events, the observed associations cannot establish whether elevated ALP preceded vascular access deterioration. Furthermore, single-time-point measurements may not adequately represent long-term biochemical exposure.

### 2.6. Statistical Analysis

Continuous variables were assessed for distribution and are presented as median and interquartile range (IQR), whereas categorical variables are reported as counts and percentages. Comparisons between patients with no cumulative vascular access burden (burden = 0) and those with cumulative burden (burden ≥ 1) were performed using the Mann–Whitney U test for continuous variables and the chi-square test or Fisher’s exact test for categorical variables, as appropriate.

Associations between clinical and laboratory variables and the cumulative vascular access burden score were assessed using Spearman’s rank correlation coefficients. The association between ALP and the presence of cumulative vascular access burden was further evaluated using binary logistic regression. Odds ratios (ORs) with 95% confidence intervals (CIs) were calculated per 10 U/L increase in ALP.

Sequential parsimonious logistic regression models were constructed to assess the stability of the association between ALP and cumulative vascular access burden while limiting the number of covariates relative to the number of outcome events. Model 1 included ALP alone; Model 2 included ALP, age, and BMI; Model 3 included ALP, age, BMI, and diabetes mellitus; and Model 4 included ALP, age, BMI, PTH, and phosphorus.

Receiver operating characteristic (ROC) curve analysis was performed to assess the discriminatory ability of ALP and PTH for the presence of cumulative vascular access burden, with results expressed as the area under the curve (AUC). Patients were also stratified according to ALP tertiles to explore the distribution of cumulative vascular access burden across increasing ALP levels.

All statistical tests were two-sided, and a *p*-value < 0.05 was considered statistically significant. Statistical analyses were performed using IBM SPSS Statistics, version 26.0 (IBM Corp., Armonk, NY, USA).

### 2.7. Ethics

The study was conducted in accordance with the Declaration of Helsinki and was approved by the Ethics Committee of the Emergency County Clinical Hospital of Craiova (approval no 23872/11 May 2026). Data collection and statistical analysis were performed after ethics committee approval. Written informed consent for the use of anonymized medical data for scientific purposes was obtained from all patients in accordance with institutional regulations.

## 3. Results

Of the 83 patients assessed for eligibility, 9 (10.8%) were excluded because of incomplete medical documentation, resulting in a final analytical cohort of 74 patients (89.2%). The included patients had a median age of 60 years (IQR 53–71) and a balanced sex distribution. Median BMI was 26.7 kg/m^2^, while median PTH and ALP levels were 256 pg/mL and 79 U/L, respectively. The main clinical and laboratory characteristics of the study cohort are summarized in Table 1.

Arteriovenous fistula was the predominant vascular access (73.0%), while 27.0% of patients had a CVC. At least one access-related complication was recorded in 37.8% of patients. Aneurysm was the most frequently documented complication (37.8%), followed by thrombosis (10.8%) and infection (5.4%). Definitive access loss occurred in 32.4% of patients (Table 2).

Patients with cumulative vascular access burden were younger (56.0 vs. 67.5 years, *p* = 0.021) and had higher BMI (31.0 vs. 25.8 kg/m^2^, *p* < 0.001). Notably, ALP levels were significantly higher in patients with cumulative access burden (92.0 vs. 78.0 U/L, *p* = 0.010), whereas PTH, calcium, phosphorus, and the calcium–phosphorus product did not differ significantly between groups (Table 3).

Among the evaluated clinical and laboratory parameters, only ALP showed a significant positive correlation with cumulative vascular access burden (Spearman’s ρ = 0.303, *p* = 0.009). No significant correlations were observed for PTH, calcium, phosphorus, calcium–phosphorus product, CRP, hemoglobin, or access vintage (Table 4).

ALP remained significantly associated with cumulative vascular access burden across all sequential models. In the fully adjusted model, each 10 U/L increase in ALP was associated with 22.6% higher odds of having a documented cumulative vascular access burden (OR = 1.226, 95% CI 1.059–1.418; *p* = 0.006), after adjustment for age, BMI, PTH, and phosphorus (Table 5).

**Figure 1:**
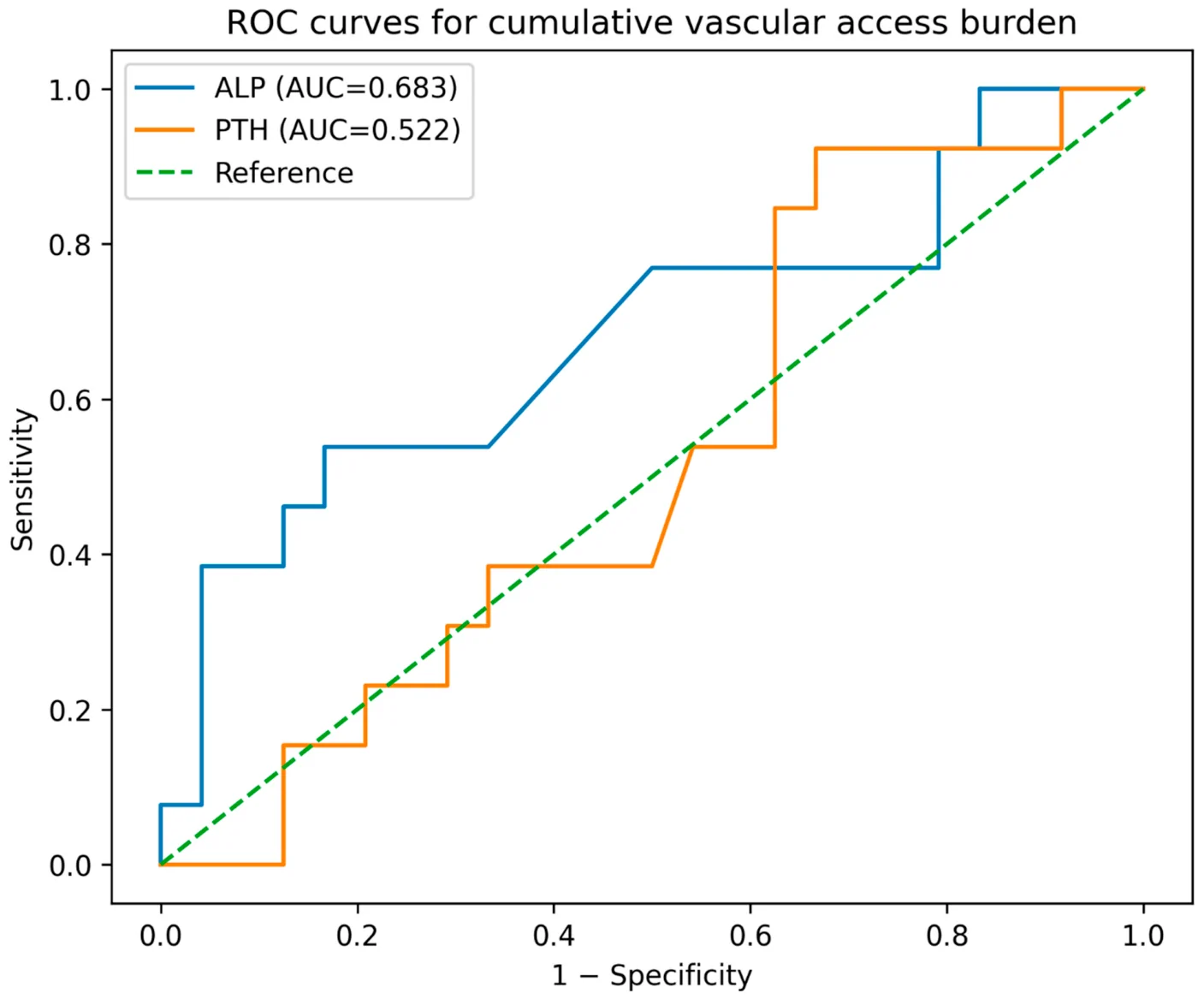
Receiver operating characteristic (ROC) curves of alkaline phosphatase (ALP) and parathyroid hormone (PTH) for cumulative vascular access burden.

**Figure 2:**
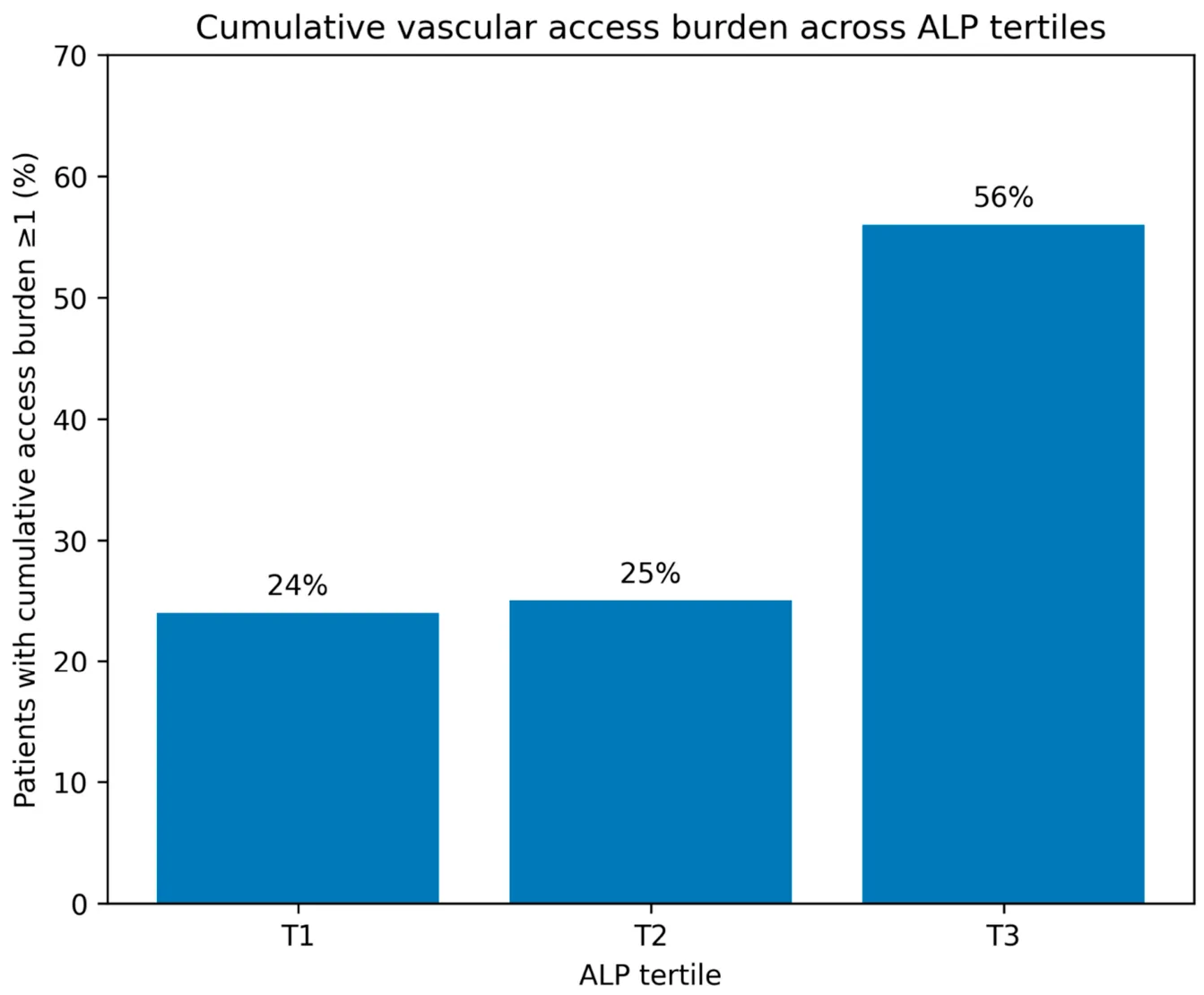
Prevalence of cumulative vascular access burden across alkaline phosphatase (ALP) tertiles, showing a higher burden in the highest ALP tertile.

ROC analysis showed modest discriminatory ability for ALP (AUC = 0.683), whereas PTH showed no meaningful discrimination (AUC = 0.522) for cumulative vascular access burden (Figure 1). The prevalence of cumulative vascular access burden was highest in the upper ALP tertile (Figure 2).

## 4. Discussion

### 4.1. Principal Findings

The main finding of this exploratory study was the association between higher alkaline phosphatase (ALP) levels and cumulative vascular access burden in patients receiving maintenance hemodialysis. In contrast, PTH, calcium, phosphorus, and the calcium–phosphorus product were not significantly associated with this outcome. ALP showed a statistically significant but modest positive correlation with cumulative vascular access burden (Spearman’s ρ = 0.303, *p* = 0.009). Given the exploratory design, relatively small sample size, and retrospective reconstruction of vascular access history, this association should be interpreted cautiously. Importantly, the observed correlation does not establish a causal relationship or demonstrate that elevated ALP can predict subsequent vascular access deterioration.

This association remained statistically significant after adjustment for selected clinical and biochemical variables. However, ALP demonstrated only modest discriminatory performance, whereas PTH showed little discriminatory value. The higher prevalence of cumulative vascular access burden among patients in the highest ALP tertile further supported the observed association.

These findings should be interpreted as an association rather than evidence of a causal relationship. Elevated ALP may identify HD patients with a greater cumulative burden of vascular access dysfunction, but it is unlikely to represent an isolated determinant of access failure. Vascular access deterioration results from several interacting processes, including neointimal hyperplasia, endothelial dysfunction, inflammation, thrombogenic mechanisms, and progressive vascular remodeling. ALP may therefore be better considered as a marker associated with an unfavorable vascular access phenotype rather than as a direct cause of access failure.

### 4.2. Comparison with the Literature

The relationship between mineral metabolism abnormalities and vascular access outcomes has been explored in several studies, although findings have varied across individual CKD–MBD biomarkers. Some investigations have reported a greater likelihood of arteriovenous fistula (AVF) intervention among patients with elevated PTH or FGF-23 concentrations and reduced 25-hydroxyvitamin D levels. In contrast, the associations involving serum calcium and phosphorus have generally been weaker or have not reached statistical significance [16,17,18]. Among these biochemical parameters, PTH has been investigated particularly extensively, with several clinical studies reporting associations between increased PTH levels and unfavorable AVF outcomes, including access failure, thrombotic complications, and reduced patency following vascular interventions [19,20,21,22].

Evidence regarding the relationship between mineral metabolism and vascular access outcomes remains heterogeneous. In the CHOICE study, which included 219 patients, Gardezi et al. observed that elevated PTH and FGF-23 concentrations were associated with a greater risk of vascular access intervention, with adjusted hazard ratios of 1.64 (95% CI 1.02–2.62) and 2.56 (95% CI 1.06–6.18), respectively. A similar association was identified for lower 25-hydroxyvitamin D levels (HR = 3.28, 95% CI 1.31–8.20) [16]. Further evidence concerning PTH was provided by Palamar et al. in a cohort of 249 hemodialysis patients. In their study, individuals who developed AVF thrombosis had higher PTH concentrations than those without thrombosis (494.6 vs. 381.5 pg/mL; *p* = 0.047) [21]. However, these observations have not been consistent across all investigations. Kubiak et al., examining 562 patients, did not identify significant relationships between conventional mineral metabolism parameters and AVF maturation failure [23]. Differences in patient characteristics, clinical settings, and the vascular access outcomes evaluated may partly explain these discrepancies. Our study approached this issue from a different perspective by examining the accumulated history of vascular access-related events rather than a single complication. Within this framework, ALP was associated with cumulative vascular access burden, while PTH, calcium, and phosphorus were not significantly associated with this outcome.

Si et al. recently identified a relationship between elevated preoperative ALP concentrations and unsuccessful arteriovenous fistula maturation [15]. Their investigation addressed a specific stage of vascular access development, whereas our analysis considered the broader clinical history of patients undergoing hemodialysis. By examining repeated interventions, permanent access losses, and the creation of additional accesses as components of a cumulative measure, we explored a different dimension of vascular access dysfunction. The association observed in our cohort therefore adds to the existing evidence on ALP, without suggesting that this enzyme represents a previously unrecognized marker of vascular access complications.

Experimental evidence provides biological support for a vascular effect of PTH. PTH has been shown to promote vascular smooth muscle cell apoptosis and calcification through endoplasmic reticulum stress-related pathways involving PERK, IRE1, CHOP, and JNK signaling [23,24]. However, clinical findings have not been uniform. In a large multicenter cohort of more than 500 patients undergoing AVF creation, FGF-23, PTH, calcium, phosphate, and several vitamin D metabolites were generally not associated with venous histological abnormalities or AVF maturation failure [18,25]. Circulating mineral markers may therefore incompletely reflect the complex vascular effects of CKD–MBD, which are also influenced by local anatomical, mechanical, metabolic, and calcification-related factors [25]. This may partly explain why PTH, calcium, and phosphorus were not associated with cumulative vascular access burden in our cohort despite their established biological relevance.

Differences between our findings and previous reports may also reflect the outcome evaluated. Most studies have examined individual endpoints such as thrombosis, stenosis, maturation failure, intervention, or loss of patency, whereas the present study considered reinterventions, definitive access losses, and additional vascular accesses as components of a cumulative outcome. This longitudinal perspective is consistent with contemporary vascular access management, which emphasizes individualized vessel preservation, contingency planning, and access succession within the ESKD Life-Plan [1].

A possible biological explanation for the association observed in our study lies in the involvement of alkaline phosphatase (ALP) in vascular calcification. Although ALP is commonly used to assess bone turnover, both tissue-nonspecific and bone-specific isoforms have also been implicated in pathological mineral deposition and vascular alterations associated with chronic kidney disease [8]. One proposed mechanism involves the enzymatic breakdown of inorganic pyrophosphate, which normally limits hydroxyapatite crystal formation. By reducing the availability of this natural inhibitor, increased ALP activity could favor calcium phosphate deposition within vascular tissues [26].

Vascular calcification represents a frequent complication among patients receiving long-term dialysis, with changes that may become more pronounced as treatment continues. In a longitudinal study involving 1489 dialysis patients monitored over four years, 86.5% experienced progression of vascular calcification. The investigators also reported a relationship between worsening coronary artery calcification and unfavorable clinical outcomes [27]. Importantly, their findings highlighted the relevance of biochemical changes over time. Average phosphorus, intact parathyroid hormone (iPTH), and FGF-23 levels during follow-up were associated with calcification progression, whereas patients who consistently maintained calcium, phosphorus, and iPTH within recommended targets were less likely to experience further calcification [27]. These findings emphasize the importance of prolonged biochemical exposure rather than isolated laboratory results. In this context, the use of single-time-point measurements in our study may have limited our ability to identify relationships involving phosphorus and PTH, potentially contributing to their lack of statistically significant associations with cumulative vascular access burden.

Evidence from the vascular access itself further supports the contribution of mineral and metabolic abnormalities to vascular remodeling. Longer AVF duration, diabetes, higher CRP, and elevated serum phosphate have been independently associated with AVF intimal hyperplasia, suggesting an interaction between metabolic factors, inflammation, mineral dysregulation, and hemodynamic stress [28]. Studies of AVF calcification similarly support a relationship between mineral and bone abnormalities and access pathology [29,30,31,32,33,34]. Although calcification has not consistently predicted AVF failure, vascular calcification and PTH have been associated with access abnormalities and poorer post-intervention patency in selected populations, suggesting that calcification represents one component of a broader multifactorial process of access deterioration.

Taken together, current evidence supports a complex relationship between CKD–MBD, vascular calcification, vascular remodeling, and vascular access outcomes. Within this context, the association between ALP and cumulative vascular access burden observed in our cohort suggests that ALP may reflect aspects of this bone–vascular interaction that are not fully represented by individual conventional mineral parameters.

### 4.3. Novelty and Clinical Interpretation

The novelty of the present study does not lie in identifying ALP as a previously unrecognized marker of vascular access dysfunction, as previous research has already reported an association between elevated preoperative ALP levels and arteriovenous fistula maturation failure [15]. Rather, the contribution of this exploratory study lies in examining the association between routinely measured total serum ALP and cumulative vascular access burden, incorporating reinterventions, definitive access losses, and additional access creation. Unlike investigations focusing on individual vascular access outcomes, our approach considers the accumulated history of access-related events at the patient level. The observed association is biologically plausible in the context of ALP involvement in vascular mineralization, although the available experimental evidence does not establish this mechanism in our cohort [8,31]. By evaluating ALP alongside conventional mineral metabolism markers, including PTH, calcium, phosphorus, and the calcium–phosphorus product, our findings provide a complementary perspective to previous research. However, the proposed composite measure remains unvalidated, and these exploratory findings require confirmation in larger prospective cohorts.

Nevertheless, ALP should not be considered an established predictor of vascular access failure. Importantly, statistical significance should not be equated with clinical predictive utility. Although ALP was significantly associated with cumulative vascular access burden in the adjusted logistic regression model (OR per 10 U/L = 1.226, 95% CI 1.059–1.418; *p* = 0.006), its modest discriminatory performance (AUC = 0.683) indicates limited ability to distinguish individual patients with and without cumulative access burden. Together with the relatively small sample size, these findings limit the clinical applicability and generalizability of the results. Therefore, ALP should be regarded as an exploratory marker that may complement established clinical and imaging-based approaches to vascular access assessment, but not as a standalone clinical prediction tool.

### 4.4. Future Perspectives

Further research involving larger patient populations and prospective, multicenter designs is needed to confirm our observations and establish a standardized approach to assessing cumulative vascular access burden. An important consideration for future investigations is the timing and frequency of ALP measurements. Repeated assessments, performed under comparable clinical and analytical conditions, may better reflect biochemical changes occurring throughout dialysis treatment than a single laboratory determination [32]. Previous longitudinal studies have demonstrated the usefulness of time-averaged mineral metabolism parameters for examining the relationship between sustained biochemical exposure and vascular calcification in dialysis populations [27]. Extending this approach to ALP could clarify whether consistently elevated concentrations or variations over time are associated with vascular access complications and the accumulation of access-related events.

Bone-specific ALP also warrants further investigation to distinguish bone-derived activity from hepatic contributions to total circulating ALP and to better define its relationship with mineralization and the bone–vascular axis.

Another important direction involves combining biochemical measurements with a more detailed evaluation of vascular access characteristics. Integrating repeated assessments of mineral metabolism, inflammatory markers, and nutritional status with vascular imaging, access flow measurements, and the evaluation of calcification, stenosis, and vessel-wall abnormalities may offer a clearer understanding of the mechanisms underlying vascular access remodeling [28]. Such an approach could help distinguish whether higher ALP levels are mainly associated with vascular calcification, neointimal hyperplasia, restenosis, thrombotic complications, or the overall accumulation of access-related events.

The potential clinical relevance of cumulative vascular access burden should also be considered within an individualized approach to vascular access planning. Current recommendations emphasize vessel preservation, contingency planning, and access succession according to patient-specific characteristics [1]. If externally validated, cumulative vascular access burden could complement this longitudinal framework by identifying patients with recurrent access-related complications and greater subsequent access requirements.

Finally, interventional studies would be required before therapeutic implications could be considered. The present findings do not establish that lowering ALP reduces vascular access complications. Further research should determine whether persistent ALP elevation primarily reflects underlying vascular vulnerability or represents a potentially modifiable CKD–MBD-related pathway involved in recurrent access deterioration.

### 4.5. Strengths and Limitations

This study has several strengths. First, vascular access outcomes were evaluated as a cumulative clinical burden rather than as isolated episodes of thrombosis, stenosis, or access failure. By incorporating reinterventions, definitive access losses, and the need for additional vascular accesses, this approach provides a broader perspective on the longitudinal history of vascular access dysfunction. Second, the association between ALP and cumulative access burden was observed across complementary statistical analyses, including group comparisons, correlation analysis, sequential multivariable models, ROC analysis, and ALP tertiles. Third, the study relied largely on routinely available clinical and laboratory parameters, supporting the potential applicability of the findings to routine hemodialysis practice. Several limitations should be acknowledged. The relatively small sample size (N = 74), including only 26 patients with cumulative vascular access burden, and the single-center design limit statistical precision and generalizability. Although sequential parsimonious logistic regression models were employed to restrict the number of covariates, the limited number of outcome events relative to the number of predictors, particularly in the fully adjusted model, increases the risk of overfitting and unstable coefficient estimates. Consequently, the observed associations, including the statistically significant association between ALP and cumulative vascular access burden, should be interpreted cautiously and regarded as exploratory rather than confirmatory. Larger prospective studies are required to assess the robustness and reproducibility of these findings.

An important limitation concerns the interpretation of total serum ALP, as bone-specific isoenzymes and hepatobiliary biochemical parameters were not systematically available, preventing differentiation between bone-derived and hepatic contributions. Furthermore, single-time-point measurements may not reflect long-term biochemical exposure. Hemodialysis duration, CKD–MBD severity, and comorbidity burden may have influenced both ALP levels and cumulative vascular access history, introducing potential residual confounding. Therefore, the observed association cannot be specifically attributed to bone–vascular mechanisms and should be interpreted cautiously. Furthermore, the retrospective data did not allow verification of uniform sampling conditions in relation to dialysis sessions, which may have introduced additional variability in biochemical measurements.

Potential confounding factors included demographic characteristics, metabolic disturbances, dialysis-related factors, and vascular comorbidities. The sequential regression models adjusted for age and BMI, with diabetes included in one model and PTH and phosphorus in the final model. However, residual confounding may persist due to factors not fully accounted for, including hemodialysis duration, cardiovascular comorbidities, inflammation, nutritional status, vascular anatomy, access characteristics, antithrombotic therapy, and cannulation practices. Furthermore, potential hepatic contributions to total ALP could not be adequately assessed. These factors may have influenced the observed association between ALP and cumulative vascular access burden.

Several potential sources of bias should be acknowledged. Selection bias may have resulted from excluding nine patients with incomplete medical records, while information bias may have arisen from the retrospective reconstruction of vascular access history. Measurement bias is possible because ALP was assessed at a single time point without standardized longitudinal measurements. Additionally, residual confounding from unmeasured clinical and vascular factors, together with differences in access surveillance and management practices, may have influenced the observed associations. These potential biases further support the exploratory interpretation of our findings.

An additional methodological limitation concerns the construction of the cumulative vascular access burden indicator. Although reinterventions, definitive access losses, and additional vascular access creation were assigned equal numerical weights, these events differ in clinical severity, consequences for future vascular access options, and healthcare resource utilization. The equal-weight approach was selected for methodological simplicity and reproducibility and should not be interpreted as indicating equivalent clinical importance. Moreover, the individual components may be influenced by surveillance practices, referral patterns, and local access-management strategies. The proposed composite indicator has not undergone external validation or formal assessment of its weighting structure. Consequently, patients with identical cumulative scores may have experienced clinically different vascular access histories. Future studies should evaluate the individual components separately and explore clinically justified weighted composite measures to improve clinical interpretability and prognostic performance.

Finally, the retrospective design and the use of a single available laboratory measurement per patient, without longitudinal biochemical monitoring, limit temporal interpretation. Because laboratory measurements were not necessarily obtained before the vascular access-related events, the observed associations cannot establish whether elevated ALP precedes access deterioration and should not be interpreted as predictive or causal. Prospective studies with serial ALP measurements are needed to clarify this temporal relationship.

## 5. Conclusions

In this exploratory study of patients receiving maintenance hemodialysis, higher ALP levels were associated with cumulative vascular access burden after adjustment for selected clinical and biochemical variables, whereas PTH, calcium, phosphorus, and the calcium–phosphorus product showed no significant associations. These findings suggest that ALP may reflect aspects of vascular vulnerability not fully captured by conventional mineral parameters. Given its modest discriminatory performance, ALP should be regarded as an exploratory marker rather than an established predictor of vascular access failure. Larger prospective studies incorporating serial and bone-specific ALP measurements are needed to validate these findings.

## Figures and Tables

**Table 1:** Baseline clinical and laboratory characteristics of the study cohort (N = 74).

Variable	N	Overall
**Age, years**	74	60.00 (53.00–71.00)
**BMI, kg/m^2^**	74	26.70 (24.00–30.46)
**Kt/V**	74	1.20 (1.11–1.40)
**Access vintage, months**	74	24.00 (12.00–48.00)
**PTH, pg/mL**	74	256.00 (199.00–530.00)
**Calcium, mg/dL**	74	8.20 (7.80–9.00)
**Phosphorus, mg/dL**	74	5.45 (4.40–7.30)
**Calcium × phosphorus, mg^2^/dL^2^**	74	45.00 (34.56–59.20)
**ALP, U/L**	74	79.00 (64.00–107.00)
**CRP, mg/L**	74	7.90 (4.00–18.00)
**Hemoglobin, g/dL**	74	10.80 (10.00–11.70)
**Female sex**	74	38 (51.4%)
**Male sex**	74	36 (48.6%)

*Data are presented as median (interquartile range) or n (%), as appropriate. ALP, alkaline phosphatase; BMI, body mass index; CRP, C-reactive protein; PTH, parathyroid hormone.*

**Table 2:** Vascular access characteristics and complications in the study cohort (N = 74).

Variable	N	n	%
**Thrombosis**	74	8	10.8
**Aneurysm**	74	28	37.8
**Access infection**	74	4	5.4
**Any recorded access complication**	74	28	37.8
**Current access: FAV**	74	54	73.0
**Current access: CVC**	74	20	27.0
**≥1 reintervention**	74	6	8.1
**≥1 definitive access loss**	74	24	32.4

*Note: CVC, central venous catheter; Access-related complications were not mutually exclusive; therefore, individual patients could have more than one documented complication.*

**Table 3:** Comparison of patients according to cumulative vascular access burden (N = 74).

Variable	N Burden = 0	Burden = 0	N Burden ≥ 1	Burden ≥ 1	*p*-Value
**Age, years**	48	67.50 (52.75–73.25)	26	56.00 (53.00–60.00)	**0.021**
**BMI, kg/m^2^**	48	25.80 (23.65–26.91)	26	31.00 (27.47–32.74)	**<0.001**
**Kt/V**	48	1.20 (1.11–1.32)	26	1.21 (1.15–1.40)	0.788
**Access vintage, months**	48	24.00 (11.50–60.00)	26	24.00 (12.00–36.00)	0.621
**PTH, pg/mL**	48	262.55 (150.75–558.50)	26	256.00 (230.00–478.90)	0.755
**Calcium, mg/dL**	48	8.20 (7.72–9.00)	26	8.20 (7.90–8.70)	0.860
**Phosphorus, mg/dL**	48	5.47 (4.35–6.85)	26	5.40 (4.70–7.40)	0.843
**Calcium × phosphorus, mg^2^/dL^2^**	48	44.34 (33.48–55.85)	26	45.50 (36.66–62.79)	0.688
**ALP, U/L**	**48**	**78.00 (63.50–81.25)**	**26**	**92.00 (79.00–161.00)**	**0.010**
**CRP, mg/L**	48	8.85 (3.88–20.25)	26	7.90 (4.20–15.10)	0.606
**Hemoglobin, g/dL**	48	10.70 (10.20–11.60)	26	11.00 (9.40–11.70)	0.896

*Note: Continuous variables are presented as median (interquartile range) and were compared using the Mann–Whitney U test. Cumulative vascular access burden was calculated as the number of reinterventions + definitive access losses + additional accesses beyond the first. ALP, alkaline phosphatase; BMI, body mass index; CRP, C-reactive protein; PTH, parathyroid hormone.*

**Table 4:** Correlations between clinical and laboratory parameters and cumulative vascular access burden (N = 74).

Variable	N	Spearman’s ρ	*p*-Value
**PTH**	74	0.122	0.299
**Calcium**	74	0.015	0.898
**Phosphorus**	74	0.018	0.877
**Calcium × phosphorus**	74	0.052	0.659
**ALP**	**74**	**0.303**	**0.009**
**CRP**	74	−0.046	0.697
**Hemoglobin**	74	0.017	0.889
**Access vintage**	74	−0.087	0.459

*Note: Spearman rank correlations were calculated in the fully imputed N = 74 dataset. ALP, alkaline phosphatase; CRP, C-reactive protein; PTH, parathyroid hormone.*

**Table 5:** Association of ALP with cumulative vascular access burden across sequential logistic regression models (N = 74).

Model	Covariates	OR per + 10 U/L ALP	95% CI	*p*-Value
**Model 1**	Unadjusted ALP	**1.149**	**1.041–1.268**	**0.006**
**Model 2**	ALP + age + BMI	**1.207**	**1.058–1.377**	**0.005**
**Model 3**	ALP + age + BMI + diabetes	**1.208**	**1.063–1.373**	**0.004**
**Model 4**	ALP + age + BMI + PTH + phosphorus	**1.226**	**1.059–1.418**	**0.006**

*Note: Binary logistic regression was performed with cumulative vascular access burden (0* vs. *≥1) as the dependent variable. ORs represent the change in odds associated with each 10 U/L increase in ALP. Models were specified as parsimonious exploratory models. ALP, alkaline phosphatase; BMI, body mass index; CI, confidence interval; OR, odds ratio; PTH, parathyroid hormone.*

## Data Availability

The data presented in this study are available on request from the corresponding author. The data are not publicly available due to patient confidentiality.
